# Environmental correlates of the forest carbon distribution in the Central Himalayas

**DOI:** 10.1002/ece3.11517

**Published:** 2024-06-18

**Authors:** Shiva Khanal, Rachael H. Nolan, Belinda E. Medlyn, Matthias M. Boer

**Affiliations:** ^1^ Forest Research and Training Center Kathmandu Nepal; ^2^ Hawkesbury Institute for the Environment Western Sydney University Richmond New South Wales Australia

**Keywords:** aboveground carbon, disturbance, environmental drivers, forest, Himalaya, soil organic carbon, terrain

## Abstract

Understanding the biophysical limitations on forest carbon across diverse ecological regions is crucial for accurately assessing and managing forest carbon stocks. This study investigates the role of climate and disturbance on the spatial variation of two key forest carbon pools: aboveground carbon (AGC) and soil organic carbon (SOC). Using plot‐level carbon pool estimates from Nepal's national forest inventory and structural equation modelling, we explore the relationship of forest carbon stocks to broad‐scale climatic water and energy availability and fine‐scale terrain and disturbance. The forest AGC and SOC models explained 25% and 59% of the observed spatial variation in forest AGC and SOC, respectively. Among the evaluated variables, disturbance exhibited the strongest negative correlation with AGC, while the availability of climatic energy demonstrated the strongest negative correlation with SOC. Disturbances such as selective logging and firewood collection result in immediate forest carbon loss, while soil carbon changes take longer to respond. The lower decomposition rates in the high‐elevation region, due to lower temperatures, preserve organic matter and contribute to the high SOC stocks observed there. These results highlight the critical role of climate and disturbance regimes in shaping landscape patterns of forest carbon stocks. Understanding the underlying drivers of these patterns is crucial for forest carbon management and conservation across diverse ecological zones including the Central Himalayas.

## Graphical abstract

Using field‐observed plot‐level carbon pool estimates from Nepal's national forest inventory, the study investigates the role of climate and disturbance in the spatial variation of aboveground carbon (AGC) and soil organic carbon (SOC) in the Central Himalayas. The climatic availability of water and energy in broad‐scale gradients combined with the fine‐scale gradients of terrain and disturbance intensity were found to influence forest carbon stocks. The results provide some important insights for forest carbon management and conservation in the Central Himalayas.

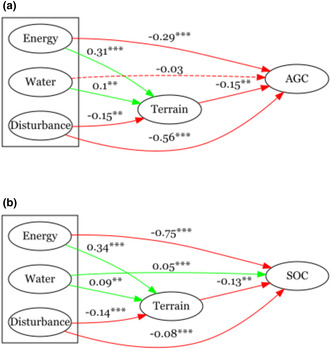

## 1 INTRODUCTION

The Himalayas are recognised as a global biodiversity hotspot (Myers et al., 2000) with a high level of endemism and the presence of priority conservation landscapes (Thompson, 2009). Recent concerns about the potential impact of climate change on vulnerable ecosystems have brought further attention to the region (Dolezal et al., 2016; Gerlitz et al., 2016; Sharma et al., 2009; Shrestha et al., 2012). The region supports diverse forest types, ranging from tropical broadleaved to alpine coniferous forests (Rawat & Lama, 2017).

Forest structures, particularly woody plant basal area, height distribution and wood density, are the primary determinants of carbon stocks (Saatchi et al., 2011) and vary with species distribution and composition (Bohn & Huth, 2017). Climate, lithology and terrain interactions impose constraints on species composition and forest structure by limiting available resources. Disturbances can alter forest structure and composition (Vlam et al., 2017), affecting carbon stocks (Zhang et al., 2014), and typically reduce forest carbon density below the climatic/edaphic potential. The environmental control of species richness over large gradients has been well studied worldwide (Sanders, 2002; Svenning et al., 2009), including in the Himalayas (Carpenter, 2005; Vetaas & Grytnes, 2002), but the relationship of species composition with forest structure, productivity and carbon storage is poorly understood. Moreover, our understanding of the relative importance of different environmental controls of forest carbon stocks across large heterogeneous landscapes such as the Central Himalayas is limited (Kohler et al., 2010; Perrigo et al., 2019).

Climatic water availability, determined by the balance between precipitation and potential evapotranspiration (PET), and climatic energy availability, characterised by temperature and solar radiation, are fundamental factors governing plant growth and adaptation (Stephenson, 1990). The interplay between these two environmental drivers shapes plant and forest ecosystems through water balance and energy‐driven physiological processes. A better quantitative understanding of these environmental controls is required to predict the spatial variation in forest carbon stocks across Nepal's highly diverse forest regions.

Variations in climate, terrain and parent material set fundamental environmental controls on forest carbon stocks. In high mountain environments, air temperature is widely considered to be the primary control of alpine treeline formation (Harsch et al., 2009; Körner, 2007), as low air temperatures and short growing seasons limit tree growth and survival beyond the treeline (Dolezal et al., 2019). In case of locations within treeline, the positive influences of both air temperature and precipitation on high‐elevation tree growth are attributed to increased moisture availability via snowmelt due to warmer spring temperatures (Wang et al., 2006). In contrast, in the subtropical regions at lower elevations, we can expect different environmental controls of forest carbon stocks, as the region has relatively high mean annual air temperature than the high mountain region (Karki et al., 2016), mostly flat terrain and highly fertile alluvium deposits (Carson, 1992). The levels of disturbance are relatively high in these lower elevation forests because of higher population density and road access (Sapkota et al., 2009; Webb & Sah, 2003).

Altitudinal gradients of mean annual air temperature vary with longitude and latitude (Champagnac et al., 2012; Cogbill & White, 1991), and affect forest species composition and structure in mountain ranges (Maharjan et al., 2022; Xu, Ma, et al., 2017). In the Central Himalayas, the elevational gradient is among the steepest on Earth and is the main cause of strong variations in a range of environmental conditions such as air temperature, precipitation, snow fraction and solar radiation. These environmental variables are physiologically important for forests, as shown by several dendrochronological studies (Gaire et al., 2014; Sigdel et al., 2020); however, their role in driving spatial variation in forest species composition and structure remains poorly quantified (Bhutia et al., 2019; Rawat & Lama, 2017). The NW‐SE orientation of the mountain range creates steep elevational gradients from south to north, overwhelming the effects of latitudinal gradients on mean annual air temperature. There can be considerable variation in elevation for a given latitude and longitude and, therefore, in the mean annual surface air temperature (Kattel et al., 2013). Additionally, the monsoon rains originating in the Bay of Bengal generate an east–west gradient in mean annual precipitation and its seasonal timing (Brunello et al., 2020), with the mid‐elevation region receiving the highest annual rainfall (Kansakar et al., 2004). Mean annual precipitation and seasonality are significant determinants of forest habitat quality, as available soil water affects species composition (Miller et al., 2021), tree growth (Eckes‐Shephard et al., 2021) and, thus, carbon storage (Hofhansl et al., 2020; Knapp et al., 2017). This geographic gradient in rainfall and air temperature significantly influences species composition and distribution within the region. Landscape‐scale variation in forest site conditions, influenced by landform, slope and aspect, is superimposed on the broad‐scale climatic gradients related to orography, longitude and latitude. The topographic relief of the Central Himalayas results in complex spatial variation in factors such as solar radiation, substrate quality, moisture availability, nutrient retention and local microclimate (Holland & Steyn, 1975; Taylor et al., 2015; Xu, Saatchi, et al., 2017; Yang et al., 2016). These topoclimatic variations shape habitats for forests with different species compositions, structures and carbon densities.

The local temperature, radiation and moisture conditions of a forest habitat are influenced by fine‐scale topographic features, broad‐scale orography and climate. Disturbances, especially those related to human land use, operate at an even finer scale, such as when people access patches to collect forest resources. These factors, operating at different spatial and temporal scales, have significant implications for forest carbon modelling across the Central Himalayas. In Nepal, fine‐resolution observations of environmental predictors, such as air temperature, soil moisture and soil depth, are unavailable. However, fine‐resolution digital elevation models (DEMs) can accurately represent terrain, which affects air temperature and moisture regimes. Using DEMs, we can derive terrain attributes that indirectly capture the variation in environmental conditions (Wilson, 2018). For instance, slope angle and aspect influence solar radiation (Kumar et al., 1997) and temperature (Sheng et al., 2009) regimes. Additionally, slope angle and slope form affect soil depth (Boer et al., 1996), drainage (Jones, 1987; Schoorl et al., 2002), snow accumulation (Jain et al., 2009), erosion (Mitas & Mitasova, 1998) and landslide risks (Pradhan & Kim, 2018). Considering the influence of topography on local climatic conditions, soil characteristics and vegetation structure over mountainous terrain (Tetzlaff et al., 2007), it is reasonable to anticipate that terrain attributes will account for a portion of the variability associated with forest carbon stocks. By combining fine‐resolution terrain attributes with coarse‐resolution gridded climate data, we may be able to capture spatial variation in environmental conditions and use that information to predict spatial variation in forest carbon stocks.

**FIGURE 1:**
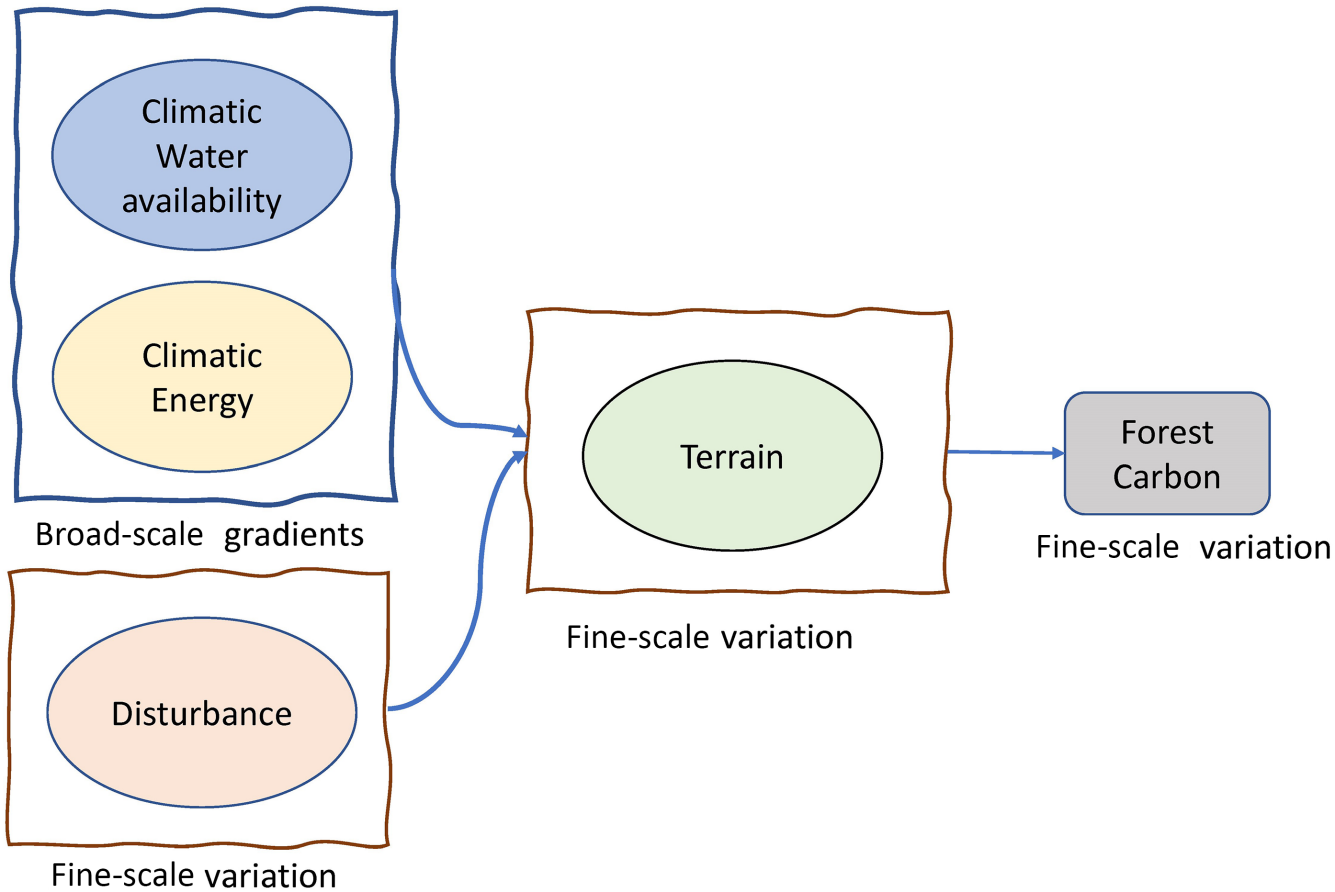
Conceptual model of controls on spatial variation in forest carbon stocks in the Central Himalayas.

The broad and fine‐scale environmental gradients expected to affect forest carbon are summarised in a conceptual model (Figure 1). Based on this conceptual model, we address the following questions:

- What are the relative influences of broad‐scale climatic variables, and fine‐scale terrain and disturbance variables in driving spatial patterns of forest carbon stocks?
- What is the most influential measured variable driving variation in forest AGC and SOC?

## 2 MATERIALS AND METHODS

### 2.1 Input data

**FIGURE 2:**
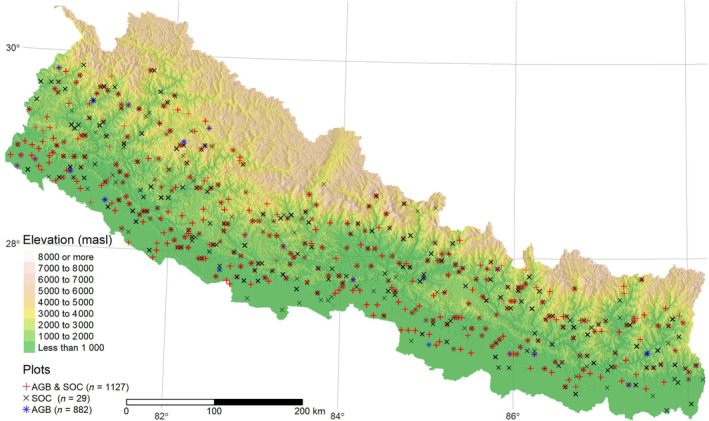
Map displaying the spatial distribution of forest inventory plots across Nepal. Three different plot types are shown: ‘+’ symbols for plots where forest aboveground biomass (AGB) and soil organic carbon (SOC) were measured, ‘x’ symbols and ‘*’ symbols for plots where only forest SOC or forest AGB was measured.

Estimates of forest carbon stocks were obtained from Nepal's national forest inventory, conducted between 2010 and 2014. We used existing field‐based estimates of two forest carbon pools: aboveground biomass (AGB) and soil organic carbon (SOC) stocks. Plot‐level estimates of forest AGB and SOC were available for 2009 and 1156 plots, respectively (Figure 2). A carbon fraction of 0.47 was used to convert forest AGB to aboveground carbon (AGC) (IPCC, 2006). Mean plot‐level AGB and SOC were 170.66 t ha^1^ and 62.30 t ha^−1^, respectively. Further descriptive statistics and full details of input data sets are presented in Table S4 and Khanal and Boer (2023), respectively.

Growing degree days (GDD) that exceed a certain temperature threshold indicate the temperature regime and duration of the forest growing season (Hanes & Schwartz, 2011; Hankin et al., 2019). In this study, a threshold of 0°C was used to calculate the GDD, given that the study area extends to cold alpine regions. Potential evapotranspiration measures the availability of climatic energy (Stephenson, 1990). Both the GDD and PET are proxies for climatic energy availability.

Proxies of terrain and disturbance variables are available at a higher spatial resolution than that of the relatively low‐resolution gridded climate datasets. Two terrain variables were used to capture fine‐scale variations in potential water and energy availability: the topographic wetness index (TWI) (Beven & Kirkby, 1979) and potential incoming solar radiation (PISR) (Lacelle et al., 2016). TWI represents fine‐scale variation in potential soil moisture availability (Muscarella et al., 2020; Rodhe & Seibert, 1999), whereas PISR represents the potential energy available as a function of slope angle, aspect, day of year, latitude and topographic shading (Stettz et al., 2019).

Disturbances particularly human‐induced ones are related to accessibility, which is particularly important for forest product collection such as tree harvesting. Forested stands closer to the road network are expected to have a higher likelihood of disturbance. In this study, we assumed the likelihood of disturbance of forest areas to be proportional to the distance to the interface with non‐forest areas. Fragmented forests have a large interface with non‐forest land use, such as settlements, and hence have high disturbance probabilities. Patch metrics from landscape ecology (McGarigal et al., 2009) were used to quantify the fragmentation of forested landscapes as a measure of the probability of disturbance. The landscape shape index (LSI) (McGarigal, 1995) provides a standardised measure of the total edge or edge density relative to the size of the landscape. Forested patches with a longer edge adjacent to non‐forest land use were assumed to be more likely to experience disturbance than intact stands in the core of forested areas.

The fragmentation of a forest area increases the length of the forest edge and thus the likelihood of disturbance. In mountainous terrain, landslides often occur where slopes are disturbed, such as near roads (Larsen & Parks, 1997), and rainfall‐triggered landslides on road construction sites are common in Nepal (McAdoo et al., 2018). Using a remotely sensed gridded data product of percentage tree cover (Hansen et al., 2013), we computed the landscape openness (LOPEN) for each 30‐m grid cell as the percentage of the area within a 1‐km buffer that exceeds 10% tree cover. Tree cover of 10% is widely accepted by the United Nations Food and Agriculture Organisation as a criterion for defining forests (FAO, 2010). LOPEN is similar to patch percentage metrics, which represents the proportion of the landscape made up of a specific patch type (Liu & Weng, 2008), and is used to quantify land‐use and land cover changes (Herzog et al., 2001). Unlike the direct use of tree cover, the openness variable represents the proportion of non‐forest areas within the surrounding landscape of each grid cell. Forest areas with high landscape openness or low tree cover can be a result of either naturally sparse forests or a reduction in tree cover due to disturbance. However, even stands with naturally low or sparse tree cover are likely to have a larger interface with non‐forest land cover, resulting in a higher probability of disturbance.

We selected predictors to represent climate, disturbance and terrain (Table 1). Details on the calculations of LSI, PISR and TWI can be found in Supporting information S1.2: Appendix S1. A summary of the descriptive statistics for input datasets is provided in Table S4.

**TABLE 1:** Description of measured variables used to inform latent variables within structural equation models to determine the drivers of forest carbon stocks. See Figure 1 for the theoretical metamodel showing the hypothesised relationships between latent variables.

Measured variable	Latent variable	Description	Units	Spatial resolution
Bio12	Water	Mean annual precipitation derived using the mean monthly precipitation (Karger et al., 2017)	mm yr^−1^	1 km
Bio15	Water	Precipitation seasonality derived using the standard deviation of the mean monthly precipitation estimates expressed as a percentage of the annual mean (Karger et al., 2017)	‐	1 km
PET	Energy	Mean annual potential evapotranspiration (Title & Bemmels, 2018). Indicates the potential evaporation when the moisture supply is unlimited.	mm yr^−1^	1 km
GDD	Energy	Sum of the mean monthly temperature greater than the base temperature (0°C) multiplied by the total number of days. Derived using Chelsa monthly temperature data (Karger et al., 2017) and growingDegDays function in the R package envirem (Title & Bemmels, 2018)	–	1 km
Bio4	Energy	Temperature seasonality (Standard deviation of the monthly mean air temperatures) (Karger et al., 2017)	–	1 km
LSI	Disturbance	Landscape shape index. An aggregation metric representing the ratio of the edge length of forest class to the minimum total edge length of a forest patch. LSI = 1 indicates maximally aggregated patches, while an increase in the index indicates an increase in the edge length, and hence decreasing aggregation. Derived for 1 km radius buffer using lsm_c_lsi function in R package landscapemetrics (Hesselbarth et al., 2019), and binary forest cover data (DFRS, 2015)	–	30 m
LOPEN	Disturbance	Landscape Openness. Using percentage tree cover data (Hansen et al., 2013), LOPEN was calculated as the percentage of grid cells covered by at least 10% tree cover within a circle of 1 km buffer distance. The field sample plot centre was the centre of the buffer.	%	30 m
PISR	Terrain	Mean daily potential incoming solar radiation (PISR) was calculated using the SAGA package (Conrad et al., 2015). The function estimates PISR based on a lumped atmospheric transmittance model. The PISR values were calculated at 30‐day intervals for a year, and the daily average was derived.	kWh m^−2^	30 m
TWI	Terrain	Topographic wetness index indicates potential on where water tends to accumulate in a landscape. A high value indicates a high potential for water accumulation due to a low slope. Derived from 30 m spatial resolution ASTER DEM (NASA et al., 2019) and SAGA package (Conrad et al., 2015)	–	30 m

### 2.2 Statistical analysis

We used structural equation modelling (SEM) to quantify the relative influence of broad and fine‐scale environmental predictors on forest carbon stocks. SEM is suitable for examining complex relationships (Fan et al., 2016; Grace, 2006) and uses latent variables to capture complex attributes that cannot be directly measured (Bollen, 2002). We employed three different latent variables: climatic water availability (mean annual precipitation (Bio12) and seasonality (Bio15)), climatic energy (mean annual PET, mean annual GDD and seasonality of mean monthly air temperature (Bio4)) and disturbance (landscape shape index (LSI) and landscape openness (LOPEN)).

Terrain attributes that determine fine‐scale variations in soil moisture availability and potential solar radiation also contribute to the spatial variation in forest carbon. SEM allows using a mediator variable (Gana & Broc, 2019) that help explain the relationship between predictor and response. In our case of examining the drivers of variation in forest carbon stocks in heterogeneous landscapes, we employed terrain as a mediator variable that lies in the causal pathway between independent (latent) variables and the dependent variables, AGC and SOC. Mediator variable, terrain was represented as latent variable with two measured variables: mean daily potential incoming solar radiation (PISR) and topographic wetness index (TWI). We designed the SEM model to examine direct effects (e.g., the path from climatic water availability to forest carbon while controlling for terrain effects), indirect effects (e.g., the path from latent variables to forest carbon considering only terrain effects) and total effects (the sum of direct and indirect effects). Additionally, simple linear models were fitted to visualise the relationships between AGC, SOC and predictor variables.

All statistical analyses were performed using R version 4.0.3 (R Core Team, 2021). Two separate SEMs for AGC and SOC were fitted using the lavaan package (Rosseel, 2012). The models were fitted using the maximum likelihood estimation with robust standard errors, and the overall model fit was assessed using the Yuan–Bentler test statistic. The model fit was evaluated using the chi‐squared test, with a non‐significant result indicating a good model fit as it suggests that there is no discrepancy between the model‐implied and the original covariance matrix. A commonly used significance cut‐off of *p* < .05 (Hu & Bentler, 1999) was applied. To further evaluate the model fit, commonly used indices such as the comparative fit index (CFI), squared root mean residual (SRMR) and root mean squared error of approximation (RMSEA) were also calculated (Fan et al., 2016; Hooper et al., 2008). The models were compared against the acceptable levels of CFI (>0.95), SRMR (<0.5) and RMSEA (0.05–0.08) (Schumacker & Lomax, 2016).

SEM is based on the covariance among variances; therefore, a key assumption of this method is that the data are multivariate normal. If this assumption is violated (e.g., skewness and outliers), it can strongly affect the covariance. To address this, the input variables were rescaled, transformed, and standardised using the DataSetScaleTransformStandardize function (Ryberg, 2017) to approximate the multivariate normality. However, some variables still violate these assumptions (Figure S1). To address this, bootstrapping was used (Gana & Broc, 2019), with 10,000 bootstraps performed to derive standard errors.

## 3 RESULTS

### 3.1 Relative influences of broad‐scale and fine‐scale drivers

**FIGURE 3:**
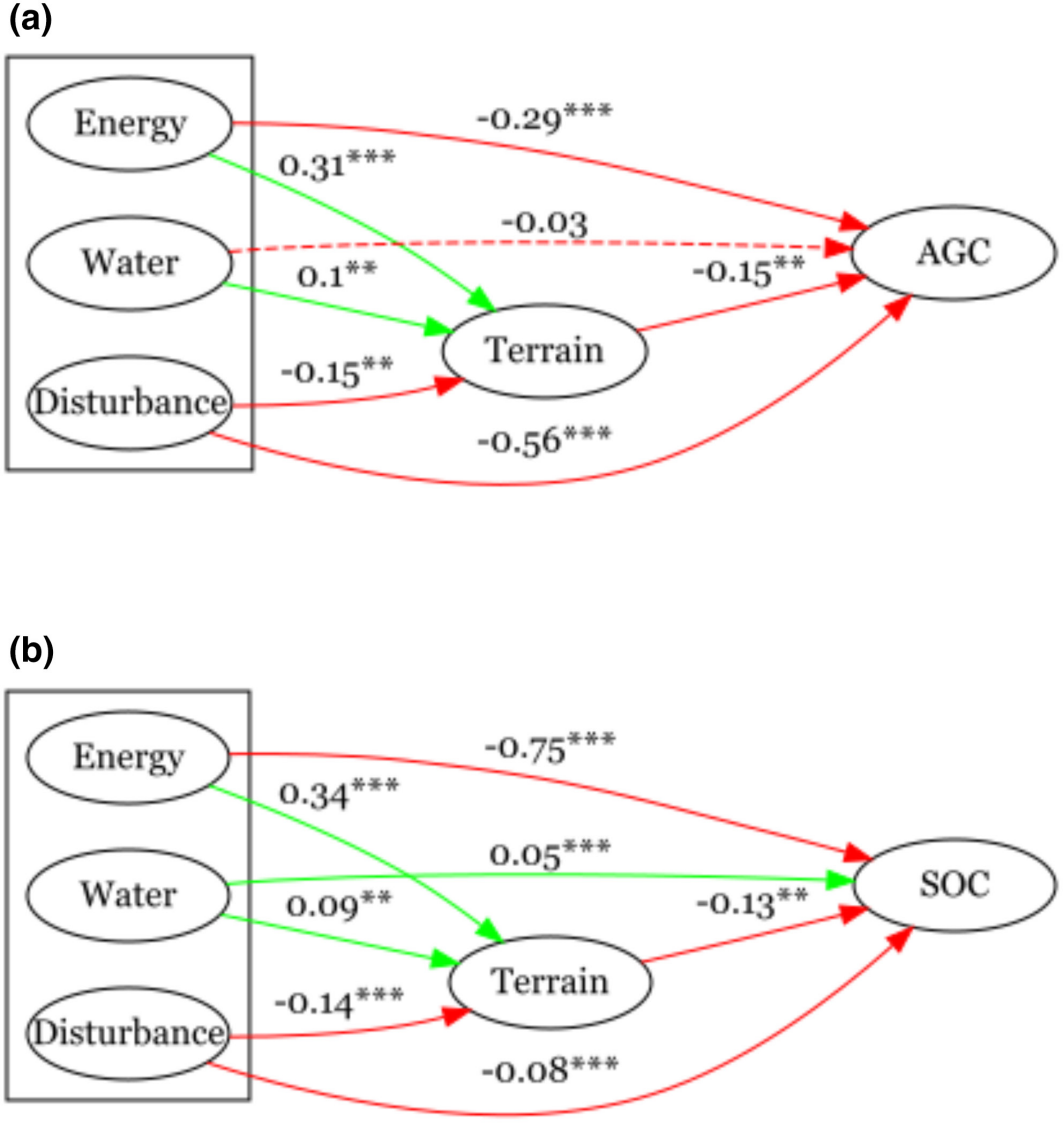
Path diagrams for structural modelling equation (SEM) models of the spatial variation in forest carbon stocks are shown in Panel a (aboveground carbon (AGC) model) and Panel b (soil organic carbon (SOC) model). Latent variables are represented by ovals enclosed in box grouping. The description of measured variables indicating latent variables can be found in Table 1. The values on arrows pointing from latent variables to observed variable indicate path regression coefficients. The significance levels are indicated by asterisks: ***(*p* < .001), **(*p* < .01), and *(*p* < .05). Positive effects are indicated by green arrows, whereas negative effects are indicated by red arrows. The dashed arrows indicate insignificant path coefficients.

The SEM for AGC explained 25% of the observed spatial variation (Figure 3a). Similarly, the SEM for SOC explained 59% of the variation (Figure 3b). The results showed that climatic energy availability was negatively correlated with forest carbon (AGC and SOC). The direct effect size of climatic energy was large, negative and significant for both forest carbon pools, with a larger impact on forest SOC compared with AGC. Variables related to climatic water availability had small but significant indirect effects on forest AGC and SOC (Figure 3). Although marginal, the direct effect size of water availability was positive for forest SOC, whereas it was negative and insignificant for forest AGC. The probability of disturbance was found to have the strongest effect on forest AGC compared with water and energy variables (Figure 3a and Table S1). Disturbance showed the strongest effect on Forest AGC. The significant total effect for the latent variable terrain suggests that it mediates the relationship between water, energy, and disturbance and forest AGC and SOC (Figure 3).

In the SEM output (Figure 3), partial mediation was observed in both the AGC and SOC models, with significant direct and indirect effects (Table S1). Partial mediation indicated that the terrain variable did not fully explain the relationship between climate and forest carbon stocks, as direct effects also played a significant role. The significant total effects, combining direct and indirect effects, further confirm the significance of partial mediation, as stated by MacKinnon et al. (2007). The partial mediation of terrain attributes on the relationship between forest carbon and climatic factors, such as energy, water and disturbance (Table S1), highlights the contribution of fine‐scale variations due to terrain superimposed on broader controls. More detailed information about the models for AGC and SOC is found in Tables S2 and S3 in Supporting information, respectively.

Among the variables, the latent variable representing disturbance probability was the most influential predictor of forest AGC, with a β coefficient of −0.556 (SE = 0.044, *p* < .001). Meanwhile, the proxy for climatic energy availability was the most influential predictor of SOC, with a β coefficient of =−0.745 (SE = 0.027, *p* < .001). A summary of the direct and total effects, including standardised coefficients, standard errors, and *p*‐values of SEMs for the AGC and SOC models, is presented in Table S1. Despite significant $\chi^{2}$ values, acceptable fit indices suggest the SEMs adequately represent the data for both AGC and SOC (details in section S1.3 of Appendix S1).

### 3.2 Relationships between measured variables and forest carbon pools

**FIGURE 4:**
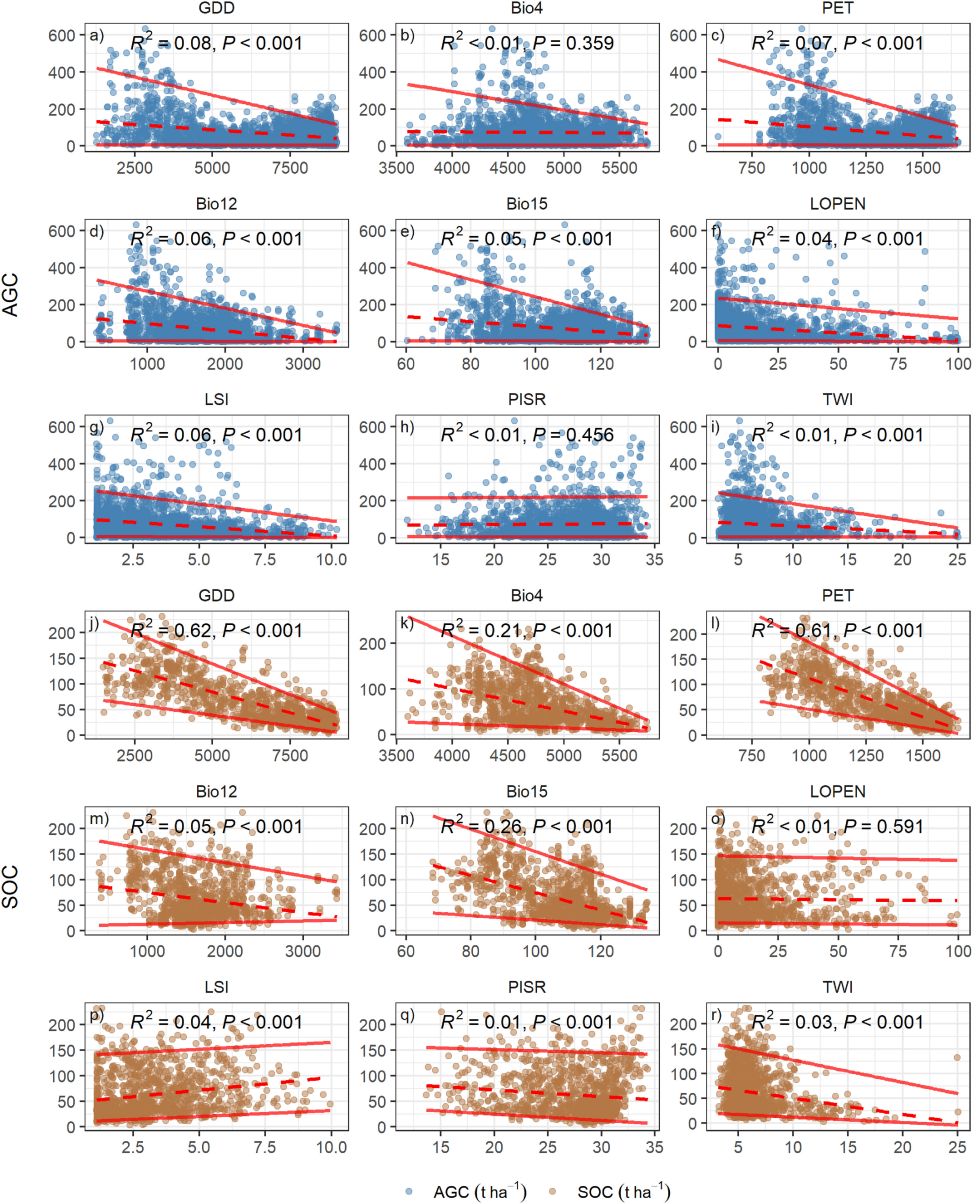
Scatterplots of forest aboveground carbon (AGC) (blue dots) and soil organic carbon (SOC) (brown dots) against each predictor used in the structural modelling equation (SEM). The solid red lines correspond to the fitted quantiles (0.05 and 0.95), and the dashed red line shows the linear fit. *R* ^2^ and *p*‐values in the panels represent linear models. AGC and SOC are on the respective *y*‐axes, and the other variables are on the *x*‐axes. The variables included mean annual growing degree days (GDD), seasonality of mean monthly air temperature (Bio4), mean annual potential evapotranspiration (PET), mean annual precipitation (Bio12), precipitation seasonality (Bio15), landscape openness (LOPEN), landscape shape index (LSI), mean daily potential incoming solar radiation (PISR) and topographic wetness index (TWI).

Our findings highlight strong relationships between climatic factors and SOC compared with AGC stocks. Univariate plots and simple linear models revealed a strong negative correlation between SOC and GDD, temperature seasonality (Bio4), and PET (Figure 4). In contrast, the relationship between GDD and forest AGC was relatively weak. However, climatic water availability (mean annual precipitation—Bio12 and precipitation seasonality—Bio15) showed a significant positive relationship with both AGC and SOC in univariate models. It is important to note that these univariate relationships may not fully capture causal effects. The observed positive direct effect of SOC and negative effect of AGC in the SEM model likely arise because SEM is a more complex model that considers all inter‐relationships between variables simultaneously.

The univariate linear model also confirmed a strong correlation between disturbance and forest AGC. Increased forest fragmentation, represented by an increase in the LSI, was associated with a decline in forest carbon. Conversely, forests with relatively low landscape openness, indicating a large proportion of tree cover, had relatively high forest carbon stock.

Although plotting forest carbon stocks (AGC and SOC) directly against terrain attributes did not show pronounced correlations (Figure 4), strong trends were observed in the upper quantiles of forest AGC and SOC for some terrain attributes. For example, despite the weak overall correlations between AGC and mean annual precipitation (Bio12), landscape openness and TWI, the upper quantiles of forest AGC showed strong trends with these attributes. Similarly, the upper quantiles of forest SOC decreased strongly with TWI, although the overall correlation between the two was weak.

## 4 DISCUSSION

This study investigated the influence of environmental factors on the spatial distribution of forest carbon stocks (AGC and SOC) in the Central Himalayas. Our findings confirm the significant effects of climate (moisture and energy availability) and disturbance on both carbon pools. The SEM explained a larger proportion of the variance in SOC compared with AGC. Additionally, SOC exhibited a stronger negative correlation with mean annual temperature (MAT), indicating a more pronounced influence of temperature on SOC storage due to slower decomposition and long‐term deposition of organic matter. While AGC also increased with decreasing mean annual air temperature, particularly at high stock levels, this relationship was fairly weak and there was substantial scatter. This suggests that tree size and stocking density, which affect biomass through volume and wood density on a finer spatial scale, are the primary drivers of forest AGC within a stand. Here, the SEM model utilised broad climatic proxies instead of direct tree structure variables, resulting in lower explained variance for AGC compared with SOC. Although these broad controls significantly influenced both carbon pools, their relative importance differed. Disturbance had the strongest negative effect on AGC, while climatic energy availability had the strongest negative effect on SOC. The significant indirect effects of terrain highlight its role in creating fine‐scale variations in habitat conditions (local climate and soil properties), ultimately influencing forest carbon stocks. These findings provide insights into the relative roles of key drivers in shaping the spatial distribution of forest carbon stocks in the Central Himalayas.

### 4.1 Broad‐scale and fine‐scale drivers

The significant negative coefficient between forest carbon stocks and climatic energy availability indicated that areas with higher carbon stocks were located in colder climates with lower GDD than in warmer climate zones. The mean annual air temperature, which decreases with elevation, can be attributed to lower plant growth rates at higher elevations. This cold air temperature limits tree growth and survival (Körner, 1998) and creates the treeline that defines the upper altitudinal limits of trees. The broad‐scale gradients of mean annual air temperature and humidity influence tree line elevation and species composition (Schickhoff et al., 2015) and, therefore, also affect forest carbon. In the Central Himalayas, the treeline elevation ranges between 3400 and 3600 metres above sea level (masl) (Schickhoff et al., 2015) and can reach even higher in some areas (Stainton, 1972). This highlights that while broad‐scale climatic gradients play a significant role, they alone cannot fully explain the variability in forest carbon stocks on a finer scale, which is influenced by the variability in forest structure due to local topoclimate and disturbance regimes.

Despite variability in forest carbon stocks within a given temperature range, it is noteworthy that plots with the highest forest carbon stocks tend to be predominantly located in areas characterised by lower temperatures. The plots with observed low AGC in the middle range of GDD correspond to middle mountain regions. These areas have a history of intense human disturbances (Brown & Shrestha, 2000; Smadja, 1992) and are frequently affected by natural disasters such as landslides (Caine & Mool, 1982). Similarly, forest SOC was relatively high in high‐elevation forest stands, consistent with a negative relationship between GDD and SOC. However, the upper‐quantile forest SOC decreased in the coldest region of the study area. This reflects that soil carbon accumulation is a long‐term process influenced by the balance between site productivity and decomposition rates, with decomposition being more sensitive than productivity. Our findings align with the observations on a global scale which shows that mature forest stands with a MAT of approximately 10°C have the highest carbon density, whereas low carbon density is found in stands with both higher and lower MAT (Liu et al., 2014). Despite the negative relationship in a linear model, the observed maximum forest carbon density stands are towards the middle MAT ranges in the Himalayas, which agrees with earlier findings (Lewis et al., 2013; Reich et al., 2014). The findings of the study agree with earlier research that shows an increase in AGC along the elevational gradient is a result of the combined effects of moisture availability and growth‐limiting air temperatures (Yang et al., 2005). Conversely, SOC increases with elevation and is related to a decrease in mean annual air temperature (Liu & Nan, 2018).

The results showed that the relationship between climatic water availability and forest SOC was significant and positive, whereas the relationship with AGC was negative. In the Central Himalayas, the major sources of soil moisture are the seasonal precipitation during the monsoon and snowpacks (Zobel & Singh, 1997). Mean annual precipitation in Nepal varies greatly, with some high‐elevation areas receiving as little as 200 mm, while others in the middle mountains receive over 5000 mm (Karki et al., 2016). Despite the limited rainfall, higher elevation areas receive seasonal snow as an additional source of moisture for plant growth (Osmaston, 1922; Singh & Singh, 1987; Trujillo et al., 2012). However, plots located at the lowest precipitation sites showed a decreasing upper quantile of forest AGC. It is important to note that the topoclimatic heterogeneity in the study area means that in some areas, rainfall increases with elevation, while in others, it decreases with elevation (Pokharel et al., 2020).

The weaker correlation between climatic water availability and forest carbon compared with climatic energy can be attributed to the effect of temperature in subalpine regions, with snow as the main source of precipitation. In these regions, temperature affects photosynthesis during the colder season but indirectly facilitates tree growth through snowmelt during winter (Huxman et al., 2003). Thus, winter temperature facilitates tree growth indirectly by increasing moisture availability through snowmelt during winter and early spring (Borgaonkar et al., 2011). As a substantial proportion of the Central Himalayan forests are located at elevations exceeding 2000 masl (DFRS, 2015), the snow fraction of precipitation is likely to be a significant determinant of forest habitat quality. The variables used to represent climatic water availability had only a marginal correlation with forest carbon, except for precipitation seasonality which showed a strong correlation with forest SOC. Forests with low SOC were located at high elevations with low annual precipitation and low precipitation seasonality. In the higher elevation regions, temperature may limit tree growth and even recruitment despite abundant precipitation as snow. Plants in the alpine region may not have water limitations but can experience a range of atmospheric effects on photosynthesis rates (Wang et al., 2017), including a decrease in the length of the growing season (Barry, 2002). The study area encompasses diverse ecoregions within the Central Himalayas (Olson et al., 2001), and the relationship between forest carbon stocks and broad climatic gradients highlights the impact of dominant climatic factors on the forests of the entire region.

As hypothesised, this study observed not only the broad‐scale interaction between climate and terrain, but also the interaction of terrain characteristics with local‐scale disturbances. The magnitude of disturbance effects on forest AGC and SOC varied, with immediate impacts on forest AGC following tree removal and slower responses in soil carbon changes. In the mountainous Central Himalayas, terrain acts as a constraint on human‐induced disturbances by limiting forest accessibility. Our findings showed that the spatiotemporal patterns of human‐induced disturbances affecting forest stands are related to topographic position and terrain attributes, as reported by Hadley (1994) and Sommerfeld et al. (2018). These results further strengthen the observation that terrain and human‐induced disturbances jointly influence forest structure and carbon content in the Himalayas, as suggested by Måren et al. (2015) and Sharma et al. (2010).

### 4.2 Measured variables and forest carbon pools

Univariate plots of the indicators of the disturbance also confirmed a significant negative relationship with forest AGC. These findings are consistent with the expectation that plots with higher disturbance likelihood had lower forest carbon. The level of forest fragmentation, as represented by the LSI, indicates a relatively high likelihood of disturbance for plots in landscapes that have a relatively high edge density. Similarly, another indicator of disturbance, landscape openness, represents the fraction of non‐forest area within the buffered region of the forest plots. Although a single or few large‐diameter trees sampled in forest inventory plots in forest stands with sparse tree canopy can result in large forest AGC estimates (Vorster et al., 2020), these occurrences are typically low. Generally, a larger proportion of non‐forested areas in a forest patch is thought to result from high‐intensity disturbance as opposed to forest patches with a closed canopy (Frolking et al., 2009). Human disturbances such as selective tree removal, which is common in Nepal, would thus reduce tree cover (Aryal et al., 2021; Shrestha et al., 2013) and, therefore, forest carbon stock. In contrast, if these sites naturally have sparse tree cover or only a fraction of the patches with tree cover, we would likely expect these sites to be adjacent to other land uses and have a higher probability of disturbance. Generally, we would expect a forest with a closed canopy to have a higher carbon density. However, in the case of forest SOC, disturbance showed a weaker relationship compared with forest AGC. The form of the relationships was as expected: the plots with maximum forest SOC occurred in locations with the lowest disturbance, forest SOC declined with the increasing disturbance and was smallest for plots in locations with the highest disturbance.

The correlation between topographic attributes and forest carbon was significant, reflecting the impact of topography on forest habitat quality through its influence on soil depth, precipitation, water redistribution and storage capacity in the mountains. Topography modifies the distribution of soil moisture availability and disturbance probabilities across the Central Himalayas (Gerlitz et al., 2016). In mountains, the formation of soil is controlled by slope and aspect, which determine the exposure to sun and wind, erosion potential and moisture retention (Price & Harden, 2013). Topography also affects the spatial variation of soil depths, which in turn controls soil moisture storage and conservation capacity (Boer et al., 1996; Williams et al., 2009). Topography affects the spatiotemporal redistribution of water by altering rainfall, snow accumulation, snowmelt and meltwater distribution (Gurung et al., 2017). Snow cover duration and other snow‐related characteristics, such as the depth and fraction of precipitation, vary depending on terrain and wind exposure. The windward slopes in the mountains receive more rainfall than the leeward slopes, creating high spatial variability in moisture availability owing to topographic complexity. Topography also affects the amount of solar radiation received across the mountainous landscape, with significant effects on forest productivity. For instance, the basal area of *Abies pindrow* in sites above 2600 masl can vary by 40% depending on the aspect (Sharma & Baduni, 2000). The potential insolation variable used in the models, which is a function of elevation, terrain slope, aspect and topographic shading (Bohner & Antonic, 2009), varies based on topographic position. The significant mediation of the relationship between forest carbon and climate by terrain most likely reflects the impact of slope angle and orientation on the spatial distributions of soil moisture (Kopecký & Čížková, 2010), solar insolation (Zhang et al., 2017) and evapotranspiration potential (Huang et al., 2019).

## 5 CONCLUSIONS

We examined how forest carbon density in the Central Himalayas is affected by key biophysical constraints such as water availability, energy availability and disturbance. We found significant variation in the relative impact of these constraints across this large and heterogeneous study area. Importantly, terrain played a crucial role by introducing fine‐scale variations in climate (water and energy availability) and disturbance. The significant effect of fine‐scale terrain on the interplay between coarse climatic variables and forest carbon density highlights the critical role of topoclimate in shaping forest carbon distribution. By incorporating these broad‐scale gradients and fine‐scale topographic variations, future studies can develop models to predict forest carbon distribution beyond the specific locations of our inventory plots. These findings suggest that the environmental controls identified here for forest carbon in the Central Himalayas may be applicable to the broader Himalayan region, warranting further investigation.

## AUTHOR CONTRIBUTIONS

**Shiva Khanal:** Conceptualization (lead); data curation (lead); formal analysis (lead); investigation (lead); methodology (lead); software (lead); writing – original draft (lead); writing – review and editing (lead). **Rachael H. Nolan:** Supervision (supporting); writing – review and editing (supporting). **Belinda E. Medlyn:** Supervision (supporting); writing – review and editing (supporting). **Matthias M. Boer:** Supervision (lead); writing – original draft (equal); writing – review and editing (equal).

## FUNDING INFORMATION

None.

## CONFLICT OF INTEREST STATEMENT

None.

## Supporting information


Appendix S1.

## Data Availability

The plot‐level estimates of forest AGB and SOC used in this study can be accessed at https://doi.org/10.6084/m9.figshare.21959636.v1.
