# Supplementary material for: Environmental correlates of the forest carbon distribution in the Central Himalayas
Source: Ecol Evol. 2024 Jun 18;14(6):e11517. doi: 10.1002/ece3.11517 (PMC11183909; doi:10.1002/ece3.11517)
Supplement: Supplementary file 1 — Appendix S1. [file ECE3-14-e11517-s001.pdf]

Supplementary material for Environmental correlates of the forest carbon distribution in the Central Himalayas

Table S1: Summary of SEMs for AGC and SOC with direct and total effects (*** p-value < 0.001; ** p-value < 0.01; * p-value < 0.05). The parameter estimates are unstandardised.

	AGC SEM	SOC SEM
	Estimate(Std.Err.)	Estimate(Std.Err.)
	Factor Loadings	
Energy		
GDD	0.96(0.02)***	0.98(0.02)***
Bio4	0.72(0.02)***	0.70(0.02)***
PET	0.93(0.02)***	0.96(0.01)***
Water		
Bio12	0.35(0.05)***	0.33(0.04)***
Bio15	1.48(0.23)***	1.59(0.18)***
Disturbance		
PTC	0.50(0.03)***	0.35(0.04)***
LSI	0.89(0.04)***	1.28(0.13)***
Terrain		
PISR	0.32(0.04)***	0.32(0.04)***
TWI	0.37(0.05)***	0.37(0.04)***
	Regression Slopes	
Terrain		
Energy	0.35(0.07)***	0.38(0.07)***
Water	0.12(0.05)*	0.11(0.04)**
Disturbance	-0.17(0.07)**	-0.16(0.04)***
AGB/SOC		
Energy	-0.29(0.04)***	-0.73(0.03)***
Water	-0.03(0.02) *	0.05(0.01)***
Disturbance	-0.56(0.05)***	-0.08(0.02)***
Terrain	-0.13(0.05)**	-0.11(0.04)**
	Constructed	
Indirect effect of Terrain on Energy	-0.04(0.02)*	-0.04(0.02)*
Indirect effect of Terrain on Water	-0.02(0.01)	-0.01(0.01)
Indirect effect of Terrain on Disturbance	0.02(0.01)	0.02(0.01)*
Total indirect effect of terrain	-0.04(0.02)	-0.04(0.02)*
Total effect (indirect + direct) of Energy	-0.05(0.02)*	0.04(0.01)**
Total effect (indirect + direct) of Water	-0.33(0.03)***	-0.77(0.02)***
Total effect (indirect + direct) of Disturbance	-0.53(0.05)***	-0.06(0.02)***
	Fit Indices	
Chi Sq	633.31(21)***	513.86(21)***
CFI	0.95	0.96
TLI	0.89	0.92
RMSEA	0.12	0.11

Rsq (PET)	0.87	0.93
Rsq (Bio12)	0.12	0.11
Rsq (Bio4)	0.52	0.49
Rsq (PISR)	0.13	0.13
Rsq (GDD)	0.92	0.97
Rsq (LOPEN)	0.25	0.12
Rsq (Terrain)	0.21	0.2
Rsq (TWI)	0.18	0.17

*p<0.05, **p<0.01, ***p<0.001

S1.1 Detailed model summary

Table S2: Standardised solution of variables in the SEM of AGC.

X	Variables	Indicator	label	B	SE	Z	p.value	Beta	Lower.CI	Upper.CI
1	Energy	GDD		0.961	0.021	46.144	0.000	0.961	0.919	1.001
2	Energy	Bio4		0.724	0.020	35.430	0.000	0.724	0.683	0.763
3	Energy	PET		0.931	0.017	53.344	0.000	0.931	0.896	0.964
4	Water	Bio12		0.351	0.049	7.123	0.000	0.352	0.243	0.436
5	Water	Bio15		1.476	0.228	6.486	0.000	1.476	1.245	2.021
6	Disturbance	LOPEN		0.502	0.031	15.993	0.000	0.503	0.440	0.563
7	Disturbance	LSI		0.891	0.038	23.543	0.000	0.891	0.820	0.969
8	Terrain	PISR		0.316	0.043	7.388	0.000	0.356	0.240	0.406
9	Terrain	TWI		0.374	0.049	7.635	0.000	0.421	0.285	0.474
10	Terrain	Energy	b1	0.349	0.070	4.988	0.000	0.310	0.213	0.490
11	Terrain	Water	b3	0.118	0.049	2.431	0.015	0.105	0.039	0.229
12	Terrain	Disturbance	b5	-0.172	0.066	-2.591	0.010	-0.153	-0.304	-0.043
13	AGB	Energy	b2	-0.285	0.040	-7.077	0.000	-0.285	-0.360	-0.202
14	AGB	Water	b4	-0.030	0.020	-1.471	0.141	-0.030	-0.078	0.002
15	AGB	Disturbance	b6	-0.555	0.046	-12.046	0.000	-0.556	-0.642	-0.462
16	AGB	Terrain	b7	-0.129	0.049	-2.634	0.008	-0.145	-0.222	-0.029
17	GDD			0.000	0.022	0.000	1.000	0.000	-0.043	0.044
18	Bio4			0.000	0.022	0.000	1.000	0.000	-0.043	0.044
19	PET			0.000	0.022	0.000	1.000	0.000	-0.043	0.044
20	Bio12			0.000	0.022	0.000	1.000	0.000	-0.043	0.045
21	Bio15			0.000	0.022	0.000	1.000	0.000	-0.044	0.045
22	LOPEN			0.000	0.022	0.000	1.000	0.000	-0.045	0.044
23	LSI			0.000	0.022	0.000	1.000	0.000	-0.044	0.042
24	PISR			0.000	0.022	0.000	1.000	0.000	-0.043	0.044
25	TWI			0.000	0.022	0.000	1.000	0.000	-0.042	0.044
26	AGB			-0.003	0.022	-0.154	0.877	-0.003	-0.047	0.041
27	Energy			0.000	0.000			0.000	0.000	0.000
28	Water			0.000	0.000			0.000	0.000	0.000
29	Disturbance			0.000	0.000			0.000	0.000	0.000
30	Terrain			0.000	0.000			0.000	0.000	0.000
31	b1b7	b1*b7		-0.045	0.021	-2.162	0.031	-0.045	-0.091	-0.009
32	b3b7	b3*b7		-0.015	0.008	-1.904	0.057	-0.015	-0.034	-0.003
33	b5b7	b5*b7		0.022	0.013	1.722	0.085	0.022	0.002	0.052

34	Total Indirect	b1*b7+b3*b7+b5*b7		-0.038	0.021	-1.806	0.071	-0.038	-0.087	-0.004
35	Total effect (indirect + direct) of Water	b3*b7+b4		-0.045	0.022	-2.074	0.038	-0.045	-0.095	-0.011
36	Total effect (indirect + direct) of Energy	b1*b7+b2		-0.330	0.033	-10.053	0.000	-0.330	-0.391	-0.263
37	Total effect (indirect + direct) of Disturbance	b5*b7+b6		-0.533	0.046	-11.634	0.000	-0.534	-0.620	-0.440

Table S3: Standardised solution of variables in the SEM of SOC.

X	Variables	Indicator	label	B	SE	Z	p.value	Beta	ci.lower	ci.upper
1	Energy	GDD		0.978	0.017	57.612	0.000	0.983	0.945	1.011
2	Energy	Bio4		0.703	0.019	37.268	0.000	0.701	0.667	0.739
3	Energy	PET		0.961	0.015	66.141	0.000	0.964	0.932	0.989
4	Water	Bio12		0.333	0.041	8.124	0.000	0.332	0.246	0.407
5	Water	Bio15		1.594	0.180	8.854	0.000	1.594	1.368	2.052
6	Disturbance	LOPEN		0.350	0.041	8.516	0.000	0.350	0.264	0.427
7	Disturbance	LSI		1.280	0.125	10.234	0.000	1.280	1.105	1.591
8	Terrain	PISR		0.321	0.041	7.771	0.000	0.360	0.246	0.408
9	Terrain	TWI		0.371	0.044	8.416	0.000	0.416	0.287	0.462
10	Terrain	Energy	b1	0.377	0.068	5.573	0.000	0.336	0.248	0.515
11	Terrain	Water	b3	0.105	0.039	2.680	0.007	0.094	0.040	0.193
12	Terrain	Disturbance	b5	-0.157	0.041	-3.824	0.000	-0.140	-0.240	-0.081
13	SOC	Energy	b2	-0.726	0.029	-25.370	0.000	-0.745	-0.780	-0.666
14	SOC	Water	b4	0.048	0.012	3.882	0.000	0.049	0.023	0.072
15	SOC	Disturbance	b6	-0.080	0.021	-3.841	0.000	-0.082	-0.124	-0.042
16	SOC	Terrain	b7	-0.114	0.042	-2.713	0.007	-0.131	-0.199	-0.033
17	GDD			0.000	0.022	0.000	1.000	0.000	-0.043	0.044
18	Bio4			0.000	0.022	0.000	1.000	0.000	-0.043	0.044
19	PET			0.000	0.022	0.000	1.000	0.000	-0.043	0.044
20	Bio12			0.000	0.022	0.000	1.000	0.000	-0.043	0.045
21	Bio15			0.000	0.022	0.000	1.000	0.000	-0.044	0.045
22	LOPEN			0.000	0.022	0.000	1.000	0.000	-0.045	0.044
23	LSI			0.000	0.022	0.000	1.000	0.000	-0.044	0.042
24	PISR			0.000	0.022	0.000	1.000	0.000	-0.043	0.044
25	TWI			0.000	0.022	0.000	1.000	0.000	-0.042	0.044
26	SOC			0.038	0.025	1.537	0.124	0.039	-0.012	0.087
27	Energy			0.000	0.000			0.000	0.000	0.000
28	Water			0.000	0.000			0.000	0.000	0.000
29	Disturbance			0.000	0.000			0.000	0.000	0.000

30	Terrain		0.000	0.000			0.000	0.000	0.000
31	b1b7	b1*b7	-0.043	0.019	-2.212	0.027	-0.044	-0.087	-0.011
32	b3b7	b3*b7	-0.012	0.007	-1.796	0.072	-0.012	-0.028	-0.002
33	b5b7	b5*b7	0.018	0.008	2.137	0.033	0.018	0.005	0.037
34	Total Indirect	b1*b7+b3*b7+b5*b7	-0.037	0.018	-2.003	0.045	-0.038	-0.080	-0.009
35	Total effect (indirect + direct) of Water	b3*b7+b4	0.036	0.012	3.047	0.002	0.037	0.010	0.057
36	Total effect (indirect + direct) of Energy	b1*b7+b2	-0.769	0.021	-36.113	0.000	-0.789	-0.811	-0.727
37	Total effect (indirect + direct) of Disturbance	b5*b7+b6	-0.062	0.019	-3.330	0.001	-0.064	-0.102	-0.029

S1.2 Description of terrain attributes

S1.2.1 Landscape shape index (LSI)

Landscape shape index (LSI) provides a standardised measure of total edge or edge density that adjusts for the size of the landscape (McGarigal 1995) and is expressed as:

$$LSI = \frac{e_i}{\min e_i} \tag{1}$$

where e_i denotes the perimeter of the developed land category, and min e_i denotes the minimum perimeter of this class

S1.2.2 Potential incoming solar radiation (PISR)

Potential incoming solar radiation (PISR) includes direct and diffuse solar radiation. A detailed description of the implemented methodology is given by (Bohner and Antonic 2009). They recognised three governing factors that affect the spatial variability of solar radiation: 1) relative orientation of the Earth in relation to the sun, 2) clouds and other atmospheric inhomogeneities, and 3) topography. PISR was derived using:

$$S^*_{S(h)} = \varsigma \cdot \frac{S_{S(h)}}{sin\theta} \cdot cos\gamma \tag{2}$$

where, $S^*_{S(h)}$ is hourly topographic direct radiation to the real land surface, ς is a binary mask (shadow = 0, non-shadow = 1), $S_{S(h)}$ is hourly direct radiation to the unobstructed horizontal surface, θ is the sun elevation angle, and $cos\gamma$ is the solar illumination angle.

S1.2.3 Topographic Wetness Index (TWI)

Topographic Wetness Index (TWI) indicates water accumulation potential. A high index value represents higher water accumulation potential, lower slope and larger contributing drainage area. TWI was derived following Beven and Kirkby (1979):

$$TWI = \ln\left(\frac{a}{\tan\beta}\right) \tag{3}$$

where, a is the Specific Catchment Area (upslope catchment area divided by the contour length along with the flow pathway), and β is the slope angle.

It represents a steady-state moisture index as the soil properties are assumed to be uniform. The parameter Specific Catchment Area (SCA) indicates the total water received, and the slope represents the expected flow of water. The index is scaled by the natural logarithm.

S1.3 SEM Diagnostics

The result of the χ^2 test showed that the estimated covariance matrix significantly differed from the actual covariance matrix for both AGC ($\chi^2(21) = 633.309$, p = 0) and SOC ($\chi^2(21) = 513.863$, p = 0) models. Although a highly significant χ^2 suggests a poor global model fit (Kline 2010), the χ^2 criterion is sensitive to sample size, as it is calculated as a function of the maximum likelihood (F_{ML}) and sample size ($\chi^2 = (n-1)F_{ML}$) (Brown 2015). For example, with more than 200 samples, χ^2 typically indicates a significant probability level (Schumacker and Lomax 2016). The fit indices of both the AGC (CFI = 0.95, SRMR = 0.057, RMSEA = 0.12) and SOC (CFI = 0.961, SRMR = 0.045, RMSEA = 0.107) models indicated a good fit. Thus, the observed range of indices within commonly accepted thresholds provided satisfactory model fits, and the models characterised the data reasonably well.

S1.4 Descriptive statistics of input data

Table S4: Basic descriptive statistic of datasets used in the model

vars	mean	sd	median	min	max	range	skew	kurtosis	se
GDD	6228.47	2016.20	6618.51	1270.50	8998.30	7727.80	-0.50	-0.85	44.66
Bio4	4760.95	411.72	4747.00	3598.04	5750.00	2151.96	-0.02	-0.37	9.12
PET	1311.86	215.67	1368.12	601.13	1654.69	1053.56	-0.50	-0.95	4.78
Bio12	1644.59	476.25	1597.00	352.00	3431.00	3079.00	0.32	0.96	10.55
Bio15	106.44	13.08	109.00	60.45	134.47	74.02	-0.52	-0.21	0.29
LOPEN	83.58	19.34	90.62	0.09	100.00	99.91	-1.82	3.50	0.43
LSI	3.51	1.93	3.13	1.11	10.21	9.10	0.77	0.00	0.04
PISR	27.31	3.81	28.48	11.88	34.31	22.43	-1.10	0.91	0.08
TWI	6.52	2.59	5.85	3.17	25.07	21.90	2.69	10.64	0.06
AGB	170.66	186.61	121.55	0.00	1477.53	1477.53	2.60	9.49	4.16
SOC	62.30	43.36	47.16	2.91	231.72	228.81	1.09	0.59	1.28

S1.5 Data transformation and test for normality

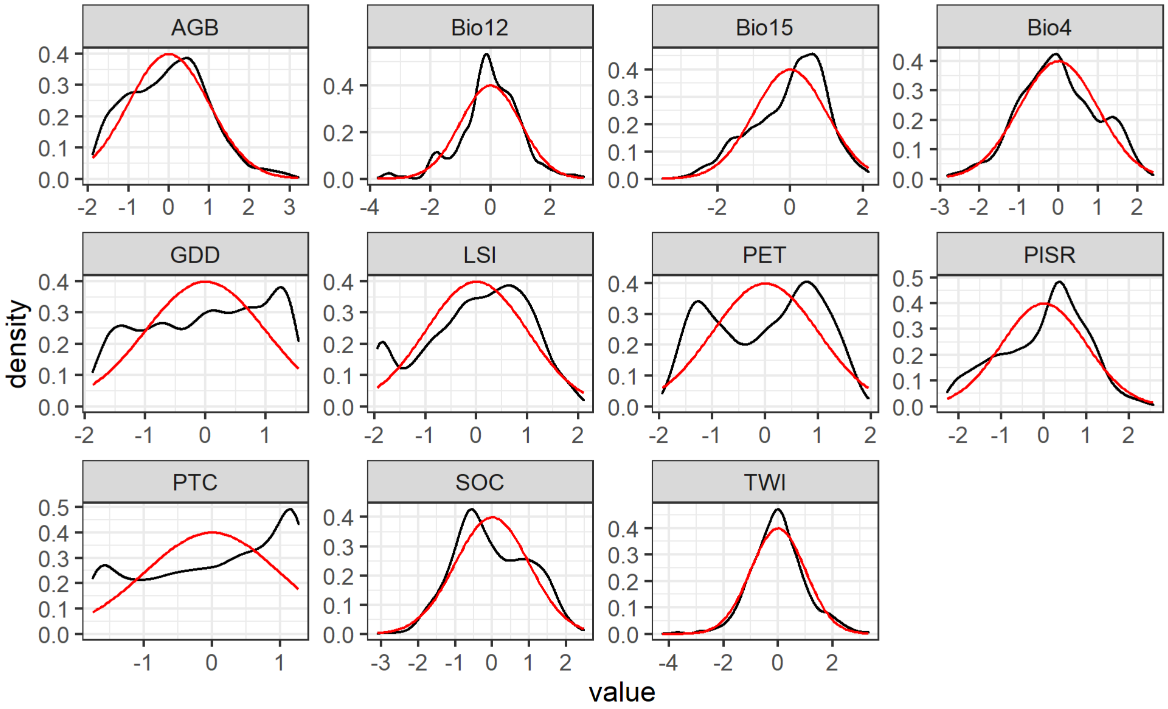

Figure S1: Univariate plots of input variables in the models. The histogram in transformed data is represented by black colour, while the normal curves are shown in red colour for comparision.

References

Beven, K. J., and M. J. Kirkby. 1979. "A Physically Based, Variable Contributing Area Model of Basin Hydrology / Un Modèle à Base Physique de Zone d'appel Variable de l'hydrologie Du Bassin Versant." *Hydrological Sciences Bulletin* 24 (1): 43–69. https://doi.org/10.1080/02626667909491834.

Bohner, J., and O. Antonic. 2009. "Chapter 8 Land-Surface Parameters Specific to Topo-Climatology." Book section. In *Developments in Soil Science*, edited by Tomislav Hengl and Hannes I. Reuter, 33:195–226. Elsevier. https://doi.org/10.1016/S0166-2481(08)00008-1.

Brown, Timothy A. 2015. *Confirmatory Factor Analysis for Applied Research.* Guilford publications.

Kline, Rex B. 2010. *Principles and Practice of Structural Equation Modeling.* 3rd ed. Methodology in the Social Sciences. New York: Guilford Press.

McGarigal, Kevin. 1995. *Fragstats: Spatial Pattern Analysis Program for Quantifying Landscape Structure.* Vol. 351. US Department of Agriculture, Forest Service, Pacific Northwest Research Station. https://doi.org/10.2737/PNW-GTR-351.

Schumacker, Randall E., and Richard G. Lomax. 2016. *A Beginner's Guide to Structural Equation Modeling.* Book. Fourth edition. New York, New York London, England : Routledge, 2016. https://doi.org/10.4324/9781315749105.
